# Chinese American and Non-Hispanic White Breast Cancer Patients’ Knowledge and Use of BRCA Testing

**DOI:** 10.3390/ijerph20043384

**Published:** 2023-02-15

**Authors:** Haocen Wang, Lei-Shih Chen, Hsin-Yi Hsiao, Suh Chen Hsiao, Tian Han, Emily Chang, Bertille Assoumou, Judy Huei-Yu Wang

**Affiliations:** 1 School of Nursing, Purdue University, West Lafayette, IN 47907, USA; 2 Department of Health Behavior, Texas A&M University, College Station, TX 77843, USA; 3 Department of Social Work, Tzu Chi University, Hualien 97074, Taiwan; 4 Department of Adult Mental Health and Wellness, Suzanne Dworak-Peck School of Social Work, University of Southern California, Los Angeles, CA 90089, USA; 5 Department of Oncology, Lombardi Comprehensive Cancer Center, Georgetown University, Washington, DC 20057, USA; 6 Veterinary Medicine & Biomedical Sciences, Texas A&M University, College Station, TX 77843, USA; 7 Department of Surgery, University of Nevada, Las Vegas, NV 89154, USA

**Keywords:** breast cancer, BRCA, genetic testing, Chinese, Non-Hispanic White

## Abstract

Breast cancer is the most commonly diagnosed cancer among Chinese American women. Knowing the BRCA1 and BRCA2 (BRCA1/2) gene mutation status can improve breast cancer patients’ health outcomes by guiding targeted treatment towards preventing breast cancer recurrence and other BRCA-related cancers. Nevertheless, it is unclear if there is a disparity in knowledge and use of BRCA testing among Chinese American breast cancer patients. This cross-sectional study investigated the possible presence of differences in the knowledge and the use of BRCA testing between Chinese American and Non-Hispanic White (NHW) breast cancer patients. We surveyed 45 Chinese American and 48 NHW adult breast cancer patients who had been diagnosed with breast cancer within the previous two years through telephone interviews. The results showed that race was not statistically related to the use of BRCA testing. BRCA testing utilization was associated with family history (*p* < 0.05) and age (*p* < 0.05). However, Chinese American participants’ understanding of BRCA testing was significantly lower than that of NHW participants (*p* = 0.030). Our findings suggest that a disparity exists in BRCA testing knowledge between Chinese American and NHW breast cancer patients. Genetic education and counseling are needed to improve BRCA testing knowledge and uptake among Chinese American breast cancer patients.

## 1. Introduction

The incidence of breast cancer, the most common cancer among women in the United States (U.S.), has continued to increase in recent years [1]. Approximately 281,550 new cases of invasive breast cancer are expected to be diagnosed, and there were over 3.8 million breast cancer survivors in the U.S. in 2021 [1]. Among all breast cancer cases, approximately 5–10% are genetically inherited [2], with 3% occurring due to variations in the BRCA1/2 genes [3]. BRCA1/2 mutation carriers can have up to a six-fold higher lifetime risk of developing breast cancer [4,5,6], and approximately a four-fold increased risk of developing a second primary contralateral breast cancer compared to women without this mutation [7].

Currently, leading agencies suggest that BRCA testing could be offered (after genetic counseling) to adults who have blood-related family members with harmful BRCA1/2 gene mutations, have a family history of BRCA-related cancers, or have a personal history that suggests an elevated risk of carrying pathogenic BRCA mutations (including but not limited to: diagnosed breast cancer at a younger age, developed second primary breast cancer, or identified as triple-negative breast cancer) [8,9,10].

For many breast cancer patients, genetic testing can be beneficial for identifying BRCA gene mutations, guiding targeted treatments, informing patients and healthcare providers to take effective action in preventing the recurrence of breast and other BRCA-related cancers, and sharing BRCA testing results with family members [7,11]. However, a discrepancy exists in knowledge about, and the utilization of, BRCA testing due to race and ethnicity [12,13,14]. Nikolaidis et al. found that young African American breast cancer patients’ knowledge about breast cancer genetics was significantly lower than Non-Hispanic White (NHW) and other breast cancer patients [15]. Studies have also shown that, among young breast cancer patients and survivors, Hispanic and African American breast cancer survivors were about 2–5.6 times less likely to utilize BRCA testing than NHWs [12,13,14].

Asian Americans are the fastest-growing racial or ethnic group in the US [16], and cancer is the leading cause of death for Asian Americans [17,18]. Chinese Americans are the largest, fastest-growing Asian subgroup in the U.S. [16]. They suffer from increasing breast cancer incidence rates, which are steadily growing by 1.1% every year [19]. The incidence rate of breast cancer among Chinese American women in the U.S. is about 82.8/100,000 [18]. 

Although the above studies report a disparity in BRCA testing utilization and knowledge between Black, Hispanic and NHW breast cancer patients, it was unclear if such a disparity also exists between Asian American and NHW breast cancer patients. The prevalence of the BRCA1 and BRCA2 mutations among Asian Americans with strong personal and/or family history of breast cancer was about 11.5% and 13%, respectively [20]. Despite the high number of Chinese American women with breast cancer, no study has been conducted to investigate the BRCA testing uptake rates and knowledge of this specific racial/ethnic subgroup. In this study, we examined the differences in BRCA testing utilization and BRCA knowledge between Chinese American and NHW female patients with breast cancer.

## 2. Materials and Methods

This study used survey data from a mixed-method research project that was designed to explore treatment decision-making among Chinese American and Non-Hispanic White breast cancer patients in Southern California [21]. The research protocol of this study was reviewed and approved by the Institutional Review Boards of Georgetown University Medical Center and the California Health and Human Services Agency.

### 2.1. Participants

Potential participants were identified through the Surveillance, Epidemiology, and End Results (SEER) cancer registry in Southern California and from community clinics. The inclusion criteria of participants were women who: (1) had been diagnosed with breast cancer within two years from the time of enrollment (from 2013 to 2015), (2) were 18 years of age or older, and (3) self-identified as Chinese or NHW. The exclusion criterion was for individuals who had a history of other cancers. Trained bilingual (Mandarin/Cantonese and English) research team members recruited participants through phone calls and in-person invitations in the community clinics.

### 2.2. Data Collection

Survey data were collected through two telephone interviews. The interviews were conducted over two weeks to decrease the burden placed upon participants and the time required to respond to survey questions. These interviews were conducted in the participant’s preferred language (Mandarin, Cantonese, or English). Of 181 eligible patients, 98 (48 Chinese Americans and 50 NHWs) consented to participate, with 93 (45 Chinese Americans and 48 NHWs) completing both interviews.

### 2.3. Survey Measures

The measures for the outcome variables (use of BRCA testing and BRCA knowledge) and covariates (family history of breast cancer and participant characteristics) are shown as follows:

Use of BRCA testing. A brief introduction to BRCA testing was first provided to the participants in the survey. Next, we included a question asking whether participants had ever utilized BRCA testing in the past (i.e., “Have you ever undergone BRCA 1/2 test?”)

BRCA knowledge. A seven-item BRCA knowledge questionnaire, developed by Peters et al. (2005) with three answer options (i.e., true, false, or do not know), was used to test participant knowledge about BRCA testing [22]. An example question was, “Can a person who does not have an altered BRCA gene still get breast cancer?” One point was given if a participant answered a question correctly, and zero points were given for incorrect answers. The range of the total BRCA knowledge score was 0–7. 

Family history of breast cancer. Participants were asked if they had any family members (i.e., parents, children, siblings, grandparents, aunts or uncles, cousins, and nieces or nephews) who had been diagnosed with breast cancer. 

Participant characteristics. Patient-reported demographic information was collected, describing their age, place of birth, educational level, marriage status, annual household income, and health insurance coverage. Additionally, the acculturation level of Chinese American participants was measured from a valid language acculturation scale using four questions that asked them to characterize their English speaking, writing, reading, and listening proficiency into five levels from 1 = “not good at all” to 5 = “very good” [23]. A mean score below 3 is considered “low language acculturation”. We also asked participants if they had any family members who had utilized BRCA testing.

### 2.4. Data Analysis

SPSS (version 26.0; Chicago, IL, USA) was used to conduct the data analysis. Descriptive statistical tests were used to analyze each participant’s use of BRCA testing, BRCA knowledge, family history of breast cancer, and demographic data. *t*-test and Chi-square tests were used to compare the differences between Chinese and NHW participants’ characteristics. Then, we applied bivariate analysis (Pearson correlation, one-way ANOVA, or cross-tabulation) to explore the associations between dependent variables (use of BRCA testing and BRCA knowledge), and independent variables (i.e., race, age, place of birth, educational level, marital status, annual household income, health insurance coverage, employment status, family history of breast cancer, the use of BRCA testing among family members, and hormone-receptor status). A binary backward logistic regression analysis was performed based on the results of the bivariate analysis to include statistically significant independent variables with the dependent variable (use of BRCA testing (*p* < 0.05) [24]. The Hosmer–Lemeshow test was used to characterize the goodness-of-fit of the binary logistic regression model, and only those variables with a *p*-value ≤ 0.2 were included in the final model following the recommendations [24]. Additionally, we used multivariable linear regression analysis with a backward elimination method (elimination criterion is *p*-value > 0.2) to investigate possible associations between the use of BRCA testing and the statistically significant independent variables based on the bivariate analysis results. Linearity, normality, and homoscedasticity assumptions for the linear regression were also examined. The annual household income was not included in the final logistic and linear regression models due to multicollinearity with the employment status. For multiple regression analysis with five and six independent variables, a sample size of 90 and 98 is required to detect a medium effect with a power of 0.8, respectively [25].

## 3. Results

### 3.1. Participant Characteristics

Table 1 summarizes participant characteristics. Among the 93 breast cancer patients in this study, 45 (48.4%) were Chinese Americans. The mean age of Chinese American participants was 57.4 years (SD = 12.35), which is significantly lower than the NHW participant’s mean age (63.9 years, SD = 11.02) (*p* = 0.009). There were significant differences between NHW and Chinese American participants regarding education levels (*p* = 0.001). While 91.7% of NHW participants had more than a high school education, only 57.8% of Chinese American participants received higher education. Additionally, the income levels between the two race groups were also significantly different (*p* = 0.002). A higher proportion of Chinese American participants (59.0%) had a lower income level (USD 50,000 or less) compared to NHW participants (21.7%).

About 68.9% and 62.5% of Chinese and NHW participants were married, respectively. All participants reported being covered by health insurance, with 60.0% of Chinese American participants and 72.9% of NHW participants being covered by private health insurance. The employment rates of Chinese and NHW participants were 44.4% and 43.8%, respectively. Approximately one-third of Chinese participants (34.1%), and half of NHW participants (50.0%) reported that they had a family history of breast cancer. Only 4 (9.3%) Chinese and 8 (17.0%) NHW participants responded that they had family members who had used BRCA testing before. A total of 53.5% of Chinese American participants were found to have a low level of language acculturation, and almost all the Chinese American participants (93.3%) were born outside the U.S. The mean English language acculturation score of Chinese American participants was 2.70 (SD = 1.08; range 1–5).

### 3.2. Use of BRCA Testing

About one-third of our participants (33.3% of Chinese Americans and 35.4% of NHWs) reported that they had utilized BRCA testing. The use of BRCA testing was not found to be significantly different between Chinese American and NHW breast cancer patients. The bivariate analyses showed that participants’ utilization of BRCA testing was significantly associated with age, annual household income level, employment status, private insurance coverage, family history of breast cancer, or family member(s) who had undergone BRCA testing (Ps < 0.05). Therefore, all these variables were included in the multivariate logistic regression model as independent variables. 

As illustrated in Table 2, the results of the logistic regression showed that participants with a family history of breast cancer were about four times more likely to utilize BRCA testing than those who did not (CI: [1.25–12.16], *p* = 0.019). Moreover, older participants were less likely to undergo BRCA testing than younger participants (age 51–64 CI: [0.04, 0.78], *p* = 0.02; age ≥ 65 CI: [0.02, 0.79], *p* = 0.03).

### 3.3. BRCA Genetic Testing Knowledge

The average BRCA genetic testing knowledge score of participants was 1.90 (SD = 1.48; range 0–7), which suggests that participants had a low level of BRCA-related knowledge. For example, none of the participants answered the following three questions correctly “all people with altered BRCA gene will get breast cancer”, “the BRCA genes cause about one-half of all breast cancers.”, and “about 1 in 10 women have an altered BRCA gene” (Table 3).

The mean BRCA knowledge score for Chinese American participants was 1.58 (SD = 1.44), which was significantly lower than the mean score of NHW participants scores (Mean = 2.2, SD = 1.47) (*p* = 0.040). Bivariate analyses showed that BRCA knowledge scores were significantly correlated with race, age, annual household income level, education level, employment status, private health insurance coverage, prior use of BRCA testing, and prior use of BRCA testing among family members (Ps < 0.05). Therefore, these variables were included in the multiple linear regression model as independent variables. 

Table 4 summarizes the final results of the multiple linear regression analysis. The variable of “use of BRCA testing” was eliminated from the final model (*p* > 0.2). Only participant race (Chinese; β = −0.25, CI: [−1.42, −0.08], *p* = 0.029) and age (≥65 years old) (β = −0.50, CI: [−2.62, −0.51], *p* = 0.004) were found to be significantly associated with BRCA knowledge score.

## 4. Discussion

In the present study, we conducted a preliminary exploration of this topic to determine if there were differences between Chinese American and NHW breast cancer patients. We did not find a significant difference in BRCA genetic testing utilization between these two groups in our sample. However, the Chinese participants had less knowledge of BRCA testing than NHW participants. 

Our results are similar to those reported in two other studies that found that race and ethnicity were not significant factors in BRCA genetic testing utilization rates among breast cancer patients [26,27], but our results differed from a study that found that Asian Americans with newly diagnosed breast cancer had a lower rate of breast cancer genetic testing for BRCA 1 and BRCA 2 genes than NHW patients [28]. Earlier research found that the cost of testing may pose a significant barrier to breast cancer genetic testing uptake for Asian Americans and other minority groups (African Americans, Native Americans, and Appalachians) [13,29]. Patients who are members of traditionally underserved populations may be less likely to discuss health issues, and be referred for genetic testing by their physicians, if they are uninsured or lack private health insurance [12,13]. The participants in this study were, however, all insured at the time of our study, and about 60% of Chinese American participants had private insurance, which may have reduced their concern with the cost of this test. 

Notably, our findings also show that, overall, both the Chinese American and NHW breast cancer patients in our sample had very limited knowledge about BRCA genetic testing. These results are different from those reported in a previous study among 206 women (mean age = 50.1 years, 85.7% were NHWs) with early-onset breast cancer that found that approximately 46% of their participants (76% of tested individuals and 45% of untested individuals) had total BRCA genetic testing knowledge scores of ≥6 out of 7 [22]. The low knowledge scores of the study sample could be explained by their age (the mean age of our sample was 60.8 years), as we found that a higher knowledge score was significantly associated with younger age in this study. Since patients who are diagnosed with breast cancer at a younger age are more likely to be carriers of BRCA mutations [30], younger breast cancer patients might be more aware of, and receive more information about, BRCA genetic testing.

Overall, our findings suggest that genetic counseling and education about BRCA genetic testing may have been inadequate for our participants. A study found that only about 62% of tested high-risk breast cancer patients had received genetic counseling [28]. A lack of critical knowledge can greatly affect a patient’s ability to make informed decisions [31]. A patient needs to fully understand the benefits and risks of BRCA testing to be able to make an informed decision that corresponds with their values [31]. To ensure that breast cancer patients receive adequate BRCA genetic testing education, more effective approaches must be identified, and comprehensive guidelines established, to promote BRCA genetic testing counseling and education.

It is worth noting that Chinese American breast cancer patients had even lower levels of knowledge than NHW breast cancer patients after analyzing participants’ age, education level, employment, insurance, and whether family member(s) had undergone BRCA testing. Previous studies also found that African Americans and Hispanics had lower levels of knowledge about breast cancer genetics and testing than NHWs [15,32,33,34,35,36]. This knowledge disparity may relate to the fact that Chinese Americans, in our sample, were almost exclusively foreign-born, and more than half (53.5%) had a low English language acculturation level (as defined above). A systematic review indicated that language and communication difficulties could negatively impact breast cancer patients’ understanding of BRCA testing [37]. Our findings suggest that there is a critical need for the creation and dissemination of culturally and linguistically appropriate BRCA genetic testing health education resources to empower Chinese American breast cancer patients to make more informed health decisions. Huang et al. found that, compared to NHW breast cancer patients, Chinese American patients had lower health literacy and greater treatment decisional conflicts, due to a lack of Chinese-speaking genetic counselors in many areas [38]. Alternative strategies, such as training Chinese-speaking community educators and providing Chinese language educational materials that are easy to access, may have the potential to increase Chinese American patients’ awareness and knowledge and facilitate currently available in-person counseling. Bilingual health education about BRCA testing should also be made available on online platforms and social media to increase Chinese Americans’ awareness of the test.

It should be made clear that this study is subject to one main limitation. Due to the challenges that are faced by researchers in recruiting Chinese breast cancer patients in a specific research timeframe and area, our sample size was small, the average age of our participants was relatively high, and all were from Southern California, which limits the scope of our findings. Despite the limitation, this study serves as a pioneer pilot study that sheds light on BRCA testing in an understudied but growing Chinese American breast cancer population. It also contributes to the limited literature in the field of precision medicine and public health. The implications of the study results may help reduce health disparities in future clinical practice. Future studies with larger and more diverse samples are needed to further determine if there are disparities in BRCA testing utilization and BRCA knowledge among Chinese breast cancer patients.

## 5. Conclusions

To conclude, this study investigated and reported the differences in BRCA genetic testing utilization and knowledge between Chinese American and NHW breast cancer patients. Our study demonstrates that there is no significant difference between the BRCA testing uptake rates of Chinese American and NHW breast cancer patients. Nevertheless, all participants were found to have low levels of BRCA testing knowledge. Chinese American participants also exhibited lower levels of BRCA knowledge than NHW participants. It is critically important that effective genetic education and counseling are created and disseminated to improve BRCA genetic testing knowledge among all breast cancer patients.

## Figures and Tables

**Table 1:** Participant characteristics.

Variable		Total	Chinese American (*n* = 45)	NHW (*n* = 48)
		*n* (%)	*n* (%)	*n* (%)
Race				
	Chinese	45 (48.4)		
	NHW	48 (51.6)		
Age ^1^				
	≤50	15 (16.1)	13 (28.9)	2 (4.2)
	51–64	45 (48.4)	22 (48.9)	23 (47.9)
	≥65	33 (35.5)	10 (22.2)	23 (47.9)
Born in the U.S.		50 (55.6)	3 (6.7)	48 (100.0)
Education				
	High school or less	23 (24.7)	19 (42.2)	4 (8.3)
	Some college	21 (22.6)	6 (13.3)	15 (31.3)
	College Graduate	31 (33.3)	15 (33.3)	16 (33.3)
	Graduate school	18 (19.4)	5 (11.1)	13 (27.1)
Marriage				
	Married	61 (65.6)	31 (68.9)	30 (62.5)
	Single/separated/divorced/widowed	32 (34.4)	14 (31.1)	18 (37.5)
Annual household Income				
	≤USD 50,000	33 (38.8)	23 (59.0)	10 (21.7)
	USD 50,001–100,000	23 (27.1)	7 (17.9)	16 (34.8)
	>USD 100,000	29 (34.1)	9 (23.1)	20 (43.5)
	Missing	8	6	2
Covered by private health insurance		62 (66.7)	27 (60.0)	35 (72.9)
Employment				
	Employed	41 (44.1)	20 (44.4)	21 (43.8)
	Unemployed	29 (31.2)	21 (46.7)	8 (16.7)
	Retire	23 (24.7)	4 (8.9)	19 (39.6)
Family History of breast cancer				
	Yes	39 (41.9)	15 (34.1)	24 (50.0)
	Unknown/unsure	1	1	0
Prior use of BRCA testing		32 (34.4)	15 (33.3)	17 (35.4)
Had family member(s) who had undergone BRCA testing				
	Yes	12 (13.3)	4 (9.3)	8 (17.0)
	Unknown/unsure	3	2	1

^1^ The mean age for Chinese Americans and NHW participants was 63.92 (SD = 11.02), and 57.42 (SD = 12.35), respectively.

**Table 2:** Binary logistic regression results of the use of BRCA testing.

Variables	Use of BRCA Testing		
	B(SE)	OR	95% CI
Age			
51–64 (verse ≤ 50)	−1.80 (0.79)	0.17 *	0.04, 0.78
≥65 (verse ≤ 50)	−2.02 (0.91)	0.13 *	0.02, 0.79
Employment			
Unemployed (versus Employed)	−0.16 (0.73)	0.32	0.09, 1.15
Retired (versus Employed)	−1.15 (0.66)	0.85	0.20, 3.56
Covered by private insurance (Yes verse No)	0.89 (0.65)	2.44	0.68, 8.71
Had family member(s) who had undergone BRCA testing (Yes versus No)	1.10 (0.76)	3.00	0.68, 13.33
Family history of breast cancer (Yes verse No)	1.37 (0.58)	3.90 *	1.25, 12.16
Cox and Snell R^2^	0.242		

* *p* < 0.05.; The Hosmer and Lemeshow Test reported a *p*-value for the model of 0.938, which indicated that the model was a good fit.

**Table 3:** Participant BRCA genetic testing knowledge.

Knowledge Question	Correct Response *n* (%)		
	Total	Chinese American	NHW
1. A person who does not have an altered BRCA gene can still get breast cancer. (True)	62 (66.7)	26 (57.8)	36 (75.0)
2. All people with an altered BRCA gene will get breast cancer. (False)	0	0	0
3. The BRCA genetic test procedure is done by giving blood or a saliva sample. (True)	45 (48.4)	19 (42.2)	26 (54.2)
4. A woman with an altered BRCA gene has a high risk of ovarian cancer. (True)	46 (49.5)	20 (44.4)	26 (54.2)
5. A father can pass an altered BRCA gene to his daughter. (True)	24 (25.8)	6 (13.3)	18 (37.5)
6. The BRCA genes cause about one-half of all breast cancers. (False)	0	0	0
7. About 1 in 10 women have an altered BRCA gene. (False)	0	0	0

**Table 4:** Multiple linear regression model of BRCA genetic testing knowledge.

Variables	BRCA Knowledge Score		
	B (SE)	β	95% CI
Race			
Chinese (versus NHW)	−0.75 (0.34)	−0.25 *	−1.42, −0.08
Age			
51–64 (versus ≤50)	−0.52 (0.43)	−0.18	−1.37, 0.34
≥65 (versus ≤50)	−1.56 (0.53)	−0.50 **	−2.62, −0.51
Education			
Some college (versus high school or less)	−0.37 (0.46)	−0.10	−1.27, 0.56
College graduate (versus high school or less)	−0.01 (0.41)	−0.00	−0.81, 0.81
Graduate school (versus high school or less)	0.09 (0.47)	0.03	−0.82, 1.05
Employment			
Unemployed (versus Employed)	−0.32(0.35)	−0.10	−1.02, 0.38
Retired (versus Employed)	0.26(0.40)	0.08	−0.54, 1.05
Covered by private insurance (Yes versus No)	0.55(0.33)	0.17	−0.10, 1.20
Had family member(s) who had undergone BRCA testing (Yes versus No)	0.79(0.41)	0.18	−0.03, 1.61
Adjusted R^2^	0.258		

β, scandalized regression coefficients. * *p* < 0.05, ** *p* < 0.01.

## Data Availability

Data sharing is available, pending the approval of both the Institutional Review Boards of Georgetown University Medical Center and the California Health and Human Services Agency.
